# Lung consolidation absorption time in 238 pediatric cases of mycoplasma pneumoniae pneumonia

**DOI:** 10.3389/fped.2025.1606834

**Published:** 2025-09-24

**Authors:** YueXu Ou, Jie Cao, Bin Qin, ZhengXiu Luo, HongQiang Du, YuanHui Duan, FengHua Chen, JiWei Zhou, YuanYuan Li, YingLan Zheng, XiaoMing Gan

**Affiliations:** ^1^ Department of General Medicine, Children’s Hospital of Chongqing Medical University, Chongqing, China; ^2^ National Clinical Research Center for Child Health and Disorders, Chongqing, China; ^3^ Ministry of Education key Laboratory of Child Development and Disorders, Chongqing, China; ^4^ China International Science and Technology Cooperation Base of Child Development and Critical Disorders, Chongqing, China; ^5^ Chongqing key Laboratory of Pediatric Metabolism and Inflammatory Diseases, Chongqing, China; ^6^ Department of Radiology, Children’s Hospital of Chongqing Medical University, Chongqing, China; ^7^ Department of Pneumology, Children’s Hospital of Chongqing Medical University, Chongqing, China; ^8^ Department of Rheumatology and Immunology, Children’s Hospital of Chongqing Medical University, Chongqing, China

**Keywords:** mycoplasma pneumoniae pneumonia, lung CT, lung consolidation volume, consolidation absorption time, bronchoalveolar lavage

## Abstract

**Objective:**

To investigate the lung consolidation absorption time and rate in children with mycoplasma pneumoniae pneumonia (MPP) and evaluate the impact of bronchoalveolar lavage (BAL) on absorption.

**Methods:**

Children hospitalized with MPP and lung consolidation in Children's Hospital of Chongqing Medical University, between January 2018 and May 2024, were included for analysis. Patients were divided into BAL and non-BAL groups. Propensity score matching (PSM) was used to adjust for baseline differences between groups, and sub-group analyses were performed to assess the effect of BAL on lung consolidation absorption speed.

**Results:**

Among 238 children with MPP and lung consolidation, females slightly outnumbered males (129 vs. 109), with a mean age of approximately 5 years. Most children received azithromycin as the first-line treatment. Lung consolidation accounted for 4.48% (IQR: 2.61%–7.35%) of the total lung volume pre-treatment, with an absorption rate of 96.08% (IQR: 88.02%–98.95%) observed during follow-up at a median interval of 17 days (IQR: 15–21 days). The median absorption speed was 2.15 cc/day (IQR: 1.23–4.01 cc/day), with complete absorption occurring within 18.96 days (IQR: 16.14–23.33 days). Comparative analysis of the BAL and non-BAL groups revealed significant differences in fever duration, hs-CRP levels, consolidation-to-total lung volume ratio at admission, follow-up intervals, and consolidation absorption speed. Following 1:1 propensity score matching (PSM) to control for confounding factors, a statistically significant but small-to-medium effect persisted, with the median absorption rate remaining higher in the BAL group (2.13 cc/day) compared to the non-BAL group (1.60 cc/day).

**Conclusions:**

Using CT scan to evaluate consolidation changes in children with Mycoplasma pneumonia, most children have 96% resolution within 2–3 weeks timeframe. Those who had a bronchoscopy may have a faster resolution rate but undertaking a flexible bronchoscopy under these circumstances is not a standard procedure in most settings.

## Introduction

Pneumonia remains one of the leading threats to children's health worldwide, with an estimated 120 million new cases of community-acquired pneumonia (CAP) in children annually (1). Mycoplasma pneumoniae (MP) is one of the most common pathogens causing CAP in children, accounting for 10%–40% of hospitalized pediatric CAP cases (2, 3). Globally, MP outbreaks occur regionally every 3–7 years, lasting 1–2 years per outbreak (4–7).

Most children with MPP normally have a favorable prognosis. However, approximately 12% require intensive care (8). Some may experience severe complications, such as multi-organ damage involving the circulatory, digestive, hematologic, and nervous systems, as well as pleural effusion, lung consolidation, pulmonary embolism, and even refractory MPP, necrotizing pneumonia (NP), bronchiolitis obliterans (BO), or fatal pneumonia. These complications can leave structural or functional lung damage, even life-threatening (9).

MPP presents with a variety of clinical symptoms, including fever, cough, and wheezing, and exhibits diverse imaging features such as bronchial wall thickening, ground-glass opacities, reticular patterns, segmental consolidation, atelectasis, and pleural effusion. A large retrospective study reported lung consolidation as the second most common imaging feature, with consolidation rates of 87.0% in patients with fever lasting ≤3 days and 88.0% in those with fever lasting >9 days (10). Huang et al. similarly found that lung consolidation occurred in 90.3% (679/752) of MPP cases, with higher incidences of refractory cases, ICU admissions, and oxygen therapy requirements compared to patients without consolidation (11). These cases also exhibited significantly prolonged fever durations, longer hospital stays, higher medical costs, and increased risks of refractory MPP, necrotizing pneumonia, and BO (11). Hence, understanding lung consolidation absorption time and speed has important clinical implication.

In recent years, advances in bronchoscopy technology have made fiberoptic bronchoscopy an essential tool in the diagnosis and treatment of respiratory diseases. Tang et al. was the first to report that bronchoalveolar lavage (BAL) could improve ventilation/perfusion matching in children with MPP and lung consolidation (12). Several other studies have demonstrated that BAL, when performed in combination with anti-infective and symptomatic treatments, can reduce pulmonary adverse sequelae and improve outcomes for children with MPP and lung consolidation (13–15). However, there is limited evidence on the long-term follow-up regarding consolidation absorption in MPP and whether BAL accelerates the absorption process.

This study aims to investigate lung consolidation absorption time in pediatric MPP patients, with a focus on the impact of BAL using CT-based quantitative measurements.

## Materials and methods

### Population

This study included pediatric patients diagnosed with MPP with lung consolidation who were hospitalized in the Department of General Medicine at the Children's Hospital of Chongqing Medical University between January 2018 and May 2024. Patients were divided into two groups based on whether they underwent BAL, the BAL group and the non-BAL group. After standardized and adequate anti-infective treatment, patients showed clinical improvement and underwent follow-up chest CT scans. This study was approved by the Ethics Committee of the Children's Hospital of Chongqing Medical University (File No: 2024-452).

### Inclusion criteria

1. Age between 1 month and 18 years; 2. Diagnosis of MPP meeting the criteria according to “Guidelines for the Diagnosis and Treatment of Mycoplasma pneumoniae in Children (2023 Edition)” (16), including fever and cough as primary clinical symptoms, combined with corresponding pulmonary imaging findings. Pathogen detection criteria included a single serum MP antibody titer ≥1:160 (PA method), a fourfold or greater increase in paired serum MP antibody titers during the disease course, or positive MP-DNA or RNA results; 3. Complete clinical and chest CT data; 4. Imaging evidence of lung consolidation on chest CT; 5. Initial and follow-up chest CT scans were performed at our hospital; 6. Follow-up CT imaging showing absorption of lung consolidation compared to initial findings, with no new consolidations observed.

### Exclusion criteria

1. Evidence of atelectasis on chest CT; 2. Presence of underlying conditions such as congenital heart disease, metabolic disorders, chromosomal abnormalities, bronchopulmonary dysplasia, immunodeficiency diseases, hematologic disorders, or tumors; 3. Recent history of severe trauma, surgery, or blood transfusion; 4. Incomplete clinical data.

### Data collection

Data were extracted from the electronic medical record system, including: General Information: gender, age; Clinical Symptoms: duration of cough and fever at admission, presence of severe pneumonia or respiratory failure; Laboratory Indicators: white blood cell count, high-sensitivity C-reactive protein (hs-CRP), and lactate dehydrogenase (LDH) levels; Treatment Details: use of second-line medications and corticosteroids; Imaging Findings: initial and follow-up lung CT measurements of lung consolidation volume, total lung volume, and consolidation location; interval between the two CT scans.

### Chest CT scanning technique and volume calculation

CT imaging was performed using GE and Siemens 128- and 256-slice scanners with the following parameters: pitch of 0.5:1, tube current of 160 mA, tube voltage of 120 kV, slice thickness and spacing of 5 mm, and a matrix of 512  ×  512. Both mediastinal and lung windows were scanned, with the mediastinal window set to a width of 450 HU and a level of 45 HU, and the lung window set to a width of 1,500 HU and a level of −600 HU.

Patients were scanned in the supine position; older children were guided to hold their breath during the scan, while younger children who could not cooperate were scanned under sedation or during natural sleep. The scan range extended from the thoracic inlet to the costophrenic angle, focusing on the region from the lung apex to the diaphragm.

**Figure 1:**
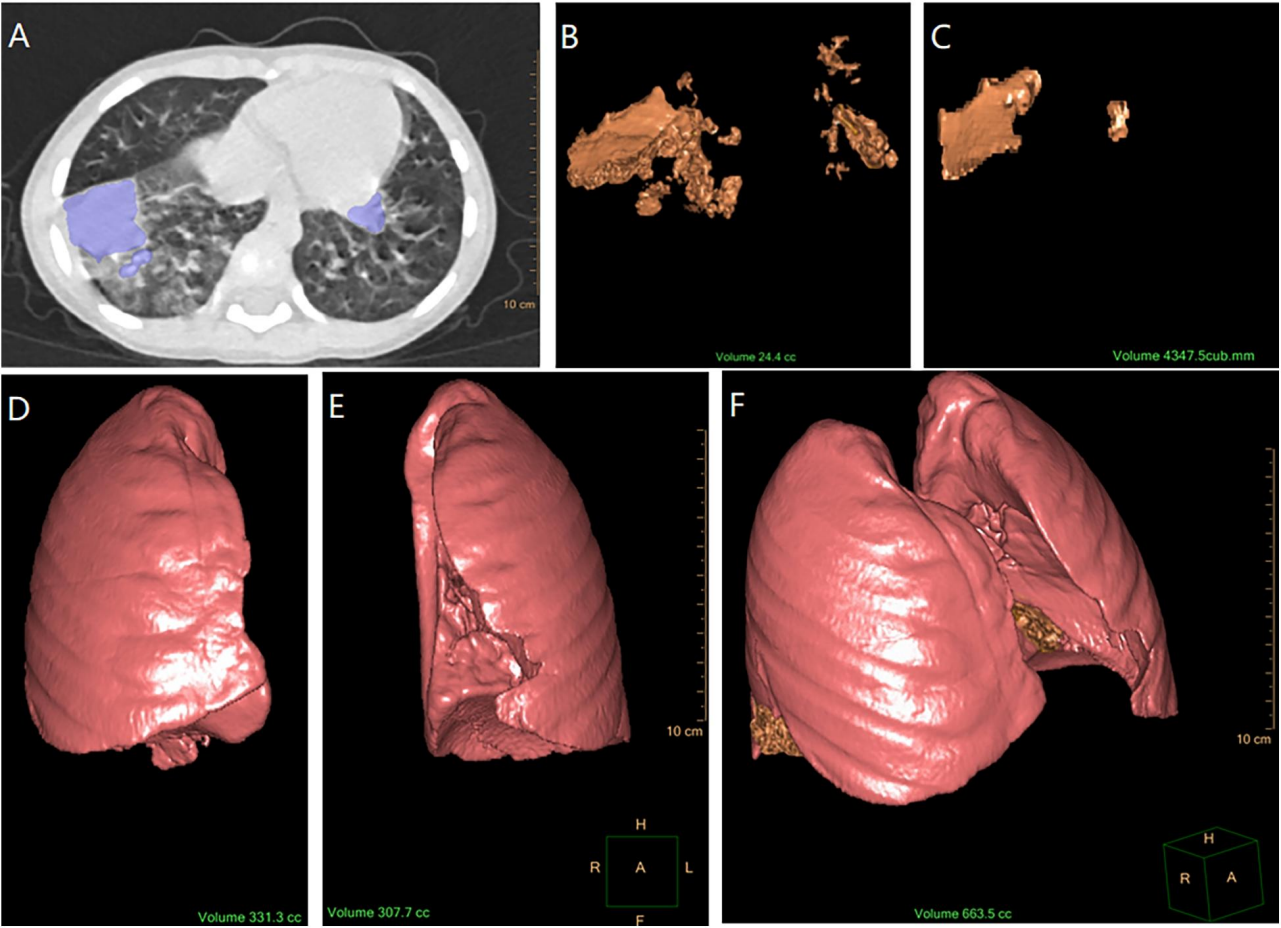
Process of calculating consolidation volume and total lung volume. **(A)** Marking the consolidation area on CT imaging (by IntelliSpace Portal software). **(B)** Calculation of initial consolidation volume using 3D reconstruction (the software automatically calculated the volume of the initial pulmonary consolidation delineated and presented it in the form of a 3D reconstruction). **(C)** Calculation of follow-up consolidation volume using 3D reconstruction (software automatical calculation). **(D)** Calculation of right lung air density tissue volume using 3D reconstruction (software automatical calculation with the bronchial structures excluded). **(E)** Calculation of left lung air density tissue volume using 3D reconstruction (software automatical calculation with the bronchial structures excluded). **(F)** Measurement of the total lung volume [The total lung volume **(F)** was obtained by adding the initial consolidation volume **(B)** to the air density volumes of the right and left lungs **(D,E)**, which is F = B + D + E].

Image analysis was conducted by two experienced radiologists. Quantitative measurements were performed using the IntelliSpace Portal software (v6.0.4.02700, Philips). The consolidation regions were manually delineated slice-by-slice in the transverse, sagittal, and coronal planes. The software automatically calculated the delineated consolidation volume using 3D reconstruction. The lung air density tissue was automatically selected, and its volume was added to the consolidation volume to calculate the total lung volume. Consolidation volumes from the initial and follow-up scans, as well as total lung volumes, were calculated, and all volumes were standardized to cubic centimeters (cc) (See Figure 1).

### BAL technique

Patients undergoing BAL met the indications outlined in the Chinese Pediatric Guidelines for Flexible Bronchoscopy (2018 Edition) (17), specifically including those who were afebrile and clinically stable following initial effective antimicrobial therapy but had persistent findings suggestive of complications like mucus plugging or extensive consolidation (e.g., diminished breath sounds, suspected airway obstruction); all cases were discussed and approved by our dedicated pediatric bronchoscopy team prior to the procedure. Preoperative evaluations included infection markers, coagulation tests, and ECG, with informed consent obtained from the guardians; should they decline BAL, a full-course antimicrobial therapy will be administered, followed by a scheduled repeat chest CT scan to assess the resolution of pulmonary lesions. BAL was performed by trained bronchoscopy specialists under intravenous anesthesia.

An appropriately sized bronchoscope was selected based on the child's age. After applying 2% lidocaine for local anesthesia at the larynx, glottis, trachea, and mainstem bronchi, the bronchoscope was inserted nasally or orally. The nasal or oral cavity, pharynx, glottis, trachea, carina, lobar, and segmental bronchi were sequentially examined. BAL was performed on the affected lung lobes or segments based on chest imaging results.

### Statistical methods

Normally distributed continuous data were expressed as mean ± standard deviation (X ± S) and compared using an independent sample *t*-test. Non-normally distributed data were expressed as medians with interquartile ranges (IQR)[P50 (P25, P75)] and analyzed with the Mann–Whitney *U* test. Categorical data were presented as frequencies and percentages (%) and compared using the chi-square test. In cases of baseline imbalance, propensity score matching (1:1) was performed to control for confounding factors before comparing the groups. Statistical significance was defined as *P* < 0.05. Statistical analysis was conducted using SPSS 24.0.

## Results

### Baseline clinical characteristics

After screening, a total of 238 cases were included (109 males and 129 females) (Table 1). Among them, 159 patients were in the BAL group, and 79 patients were in the non-BAL group. The age range was 1 month and 24 days to 13 years and 1 month, with a mean age of 5 years and 11 months ± 2 years and 6 months. At admission: Cough duration was 7 days [interquartile range (IQR): 5, 9 days]; Fever duration was 6 days (IQR: 4, 7 days); 83 patients (34.87%) were diagnosed with severe pneumonia; 18 patients (7.56%) experienced respiratory failure. Laboratory findings at admission: White blood cell (WBC) count: 6.98 × 10⁹/L (IQR: 5.47 × 10⁹/L, 8.7 × 10⁹/L); High-sensitivity C-reactive protein (hs-CRP): 12.16 mg/L (IQR: 5.02 mg/L, 23.65 mg/L); Lactate dehydrogenase (LDH): 304 U/L (IQR: 263 U/L, 355 U/L).

**Table 1:** Baseline clinical characteristics of children with MPP and pulmonary consolidation: comparison between BAL and Non-BAL groups.

Variables	Total (*n* = 238)	BAL group (*n* = 159)	Non-BAL group (*n* = 79)	*P*
Age (days)	2,162.44 ± 925.52	2,285.01 ± 899.77	1,915.75 ± 933.02	0.968
Male/female	109/129	69/90	40/39	0.359
Cough duration (day)	7 (5, 9)	7 (5.5, 9)	7 (5, 8)	0.168
Fever duration at admission (day)	6 (4, 7)	7 (5, 8)	6 (3, 7)	0.026
Severe pneumonia [*n*, (%)]	83 (34.87)	62 (38.99)	21 (26.58)	0.081
Respiration failure [*n*, (%)]	18 (7.56)	12 (7.54)	6 (7.59)	1.000
WBC (*10^9^/L)	6.98 (5.47, 8.70)	6.91 (5.50, 8.60)	7.32 (5.60, 9.14)	0.656
hs-CRP (mg/L)	12.16 (5.02, 23.65)	13.22 (5.35, 25.5)	9 (2.67, 17.2)	0.026
LDH (U/L)	304.00 (263.00, 355.00)	303 (261.50, 358.00)	304 (263.50, 348.50)	0.791
Second-line medication [*n*, (%)]	36 (15.12)	21 (13.20)	15 (18.98)	0.327
Corticosteroids use [*n*, (%)]	55 (23.10)	38 (23.89)	17(21.51)	0.805

Data are presented as median (interquartile range) or *n*(%). Mann–Whitney *U* test was used to compare nonnormally distributed continuous variables and Chi-square test was used to compare categorical variables between the two groups.

MPP, mycoplasma pneumoniae pneumonia; BAL, bronchoalveolar lavage; WBC, white blood cell; CRP, C-reactive protein; LDH, lactate dehydrogenase.

Treatment details: 36 patients (15.12%) received second-line medications (doxycycline or levofloxacin), all cases occurring in 2023 or later; 55 patients (23.10%) were treated with corticosteroids (methylprednisolone sodium succinate).

### Imaging characteristics

#### Lung consolidation features

89 patients (37.39%) had consolidation in the left lung; 117 patients (49.15%) had consolidation in the right lung; 32 patients (13.44%) had consolidation in both lungs. Initial lung consolidation volume: 40.35 cc (IQR: 24.35 cc, 76.07 cc); Total lung volume: 934.10 cc (IQR: 707.30 cc, 1,301.00 cc); Follow-up consolidation volume: 1.594 cc (IQR: 0.409 cc, 4.367 cc); Interval between scans: 17 days (IQR: 15, 21 days) (Table 2).

**Table 2:** Comparison of baseline radiological characteristics of children with MPP and pulmonary consolidation between BAL and Non-BAL groups.

Variables	Total (*n* = 238)	BAL group (*n* = 159)	Non-BAL group (*n* = 79)	*P*
Initial consolidation volume (cc)	40.35 (24.35, 76.07)	49.5 (31.2, 91.9)	27.8 (19.7, 27.8)	<0.001
Left lung consolidation [*n*, (%)]	89 (37.39)	61 (38.36)	28 (35.44)	
Right lung consolidation [*n*, (%)]	117 (49.15)	83 (52.20)	34 (43.03)	
Bilateral lung consolidation [*n*, (%)]	32 (13.44)	15 (9.43)	17 (21.51)	
Total lung volume (cc)	934.10 (707.30, 1,301.00)	1,000.7 (748.45, 1,351.55)	846.00 (670.05, 1,199.75)	0.026
Consolidation proportion (% of total lung volume)	4.48 (2.61, 7.35)	5.29 (2.95, 8.59)	3.40 (2.10, 5.09)	<0.001
Follow-up consolidation volume (cc)	1.594 (0.409, 4.367)	1.584 (0.455, 5.550)	1.714 (0.270, 3.595)	0.326
Consolidation absorption rate (%)	96.08 (88.02, 98.95)	96.47 (89.38, 99.00)	93.75 (84.75, 98.63)	0.216
Interval between scans (days)	17 (15, 21)	18 (15.5, 21.5)	16 (14, 19)	0.005
Consolidation absorption speed (cc/day)	2.15 (1.23, 4.01)	2.53 (1.48, 4.47)	1.59 (0.93, 2.73)	<0.001
Complete absorption time (days)	18.96 (16.14, 23.33)	19.68 (17.04, 23.82)	17.64 (15.52, 21.64)	0.006

BAL, bronchoalveolar lavage; cc, cubic centimeters.

Consolidation proportion = initial consolidation volume/total lung volume. The median consolidation proportion was 4.48% (IQR: 3.40%, 5.29%). Consolidation absorption = (initial consolidation volume—follow-up consolidation volume)/initial consolidation volume. The median consolidation absorption rate was 96.08% (IQR: 88.02%, 98.95%). Consolidation absorption speed = (initial consolidation volume-follow-up consolidation volume)/interval days. The median absorption speed was 2.15 cc/day (IQR: 1.23 cc/day, 4.01 cc/day). Complete absorption time = initial consolidation volume/absorption speed. The median time for complete consolidation absorption was 18.96 days (IQR: 16.14, 23.33 days).

#### Comparison between BAL and non-BAL groups

Significant differences were observed between the BAL group (159 cases) and the non-BAL group (79 cases) in terms of fever duration, hs-CRP levels, consolidation proportion, interval between scans, and consolidation absorption speed (all *P* < 0.05).

**Figure 2:**
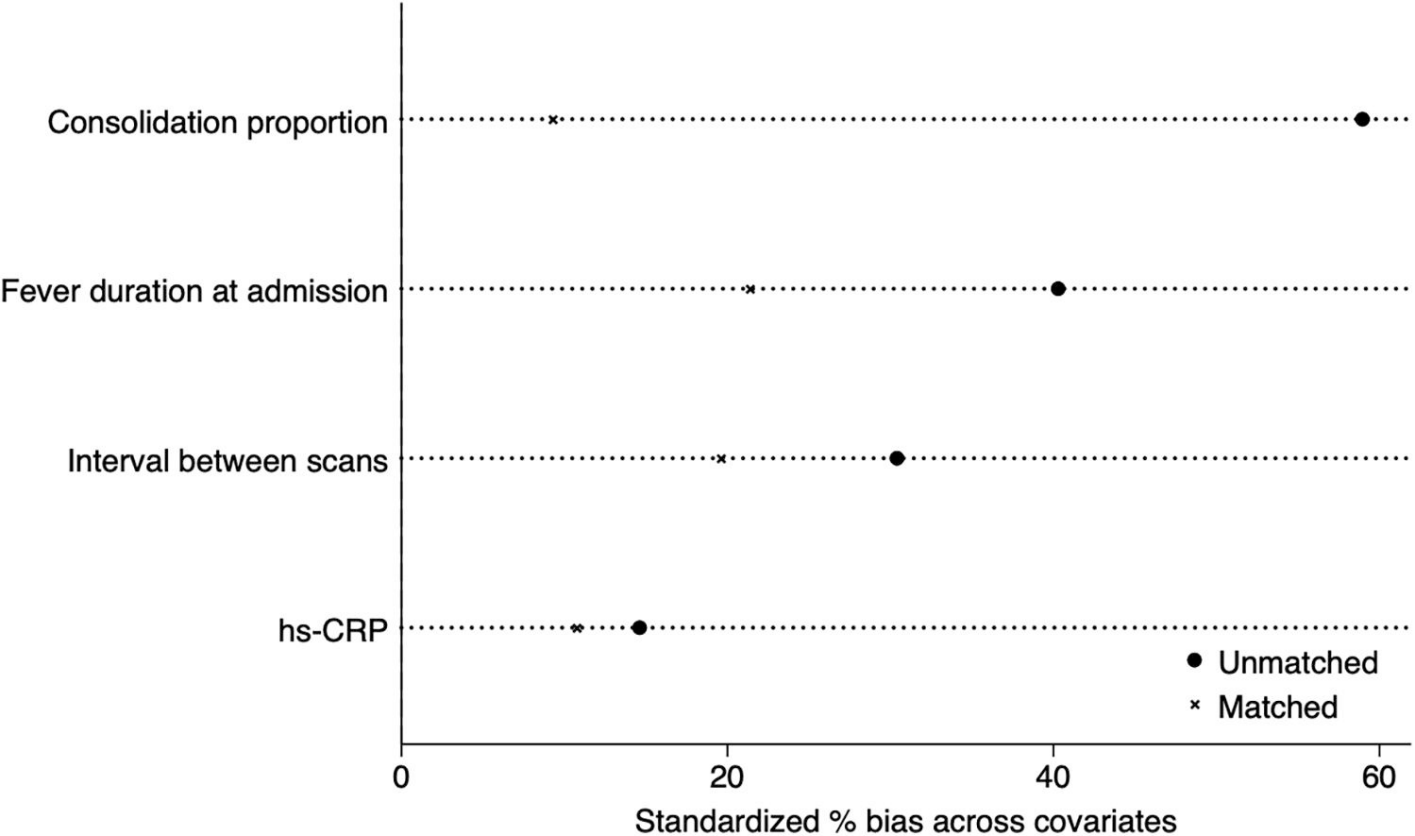
Propensity-score test demonstrating standardized bias before and after PSM.

After applying 1:1 propensity score matching to eliminate the baseline differences (including age, gender, cough duration, severe pneumonia percentage, respiration failure percentage, fever duration, WBC, hs-CRP levels, consolidation proportion, and scan interval), 76 patients remained in each group. Comparison of consolidation absorption speed between the matched groups still showed statistically significant differences (*P* < 0.05) after matching (Table 3). To increase PSM transparency, we added propensity score test (Figure 2) to visually demonstrate standardized percentage bias across significant covariates before and after PS-matching.

**Table 3:** Key characteristics between BAL and Non-BAL groups before and after PSM.

Variables	Before matching		P	After matching		P
	BAL group (*n* = 159)	Non-BAL group (*n* = 79)		BAL group (*n* = 76)	Non-BAL group (*n* = 76)	
Consolidation proportion (%)	5.29 (2.95, 8.59)	3.40 (2.10, 5.09)	0.000	4.00 (2.00, 6.00)	3.00 (2.00, 5.00)	0.200
Fever duration at admission (days)	7 (5, 8)	6 (3, 7)	0.026	6 (3, 7)	6 (3, 7)	0.716
hs-CRP (mg/L)	13.22 (5.35, 25.5)	9 (2.67, 17.2)	0.026	12.6 (5.36, 23.86)	8.86 (2.30, 19.88)	0.104
Interval between scans (days)	18 (15.5, 21.5)	16 (14, 19）	0.005	17 (15, 20)	16 (14.5, 19.5)	0.304
Consolidation absorption speed (cc/day)	2.53 (1.48, 4.47)	1.59 (0.93, 2.73)	0.000	2.13 (1.26, 3.36)	1.60 (0.93, 2.866)	0.040

BAL, bronchoalveolar lavage; cc, cubic centimeters; CRP, C-reactive protein; PSM, propensity score match.

The median consolidation absorption speed was 2.13 cc/day in the BAL group and 1.60 cc/day in the non-BAL group. The Shapiro–Wilk test indicated that consolidation speed did not follow a normal distribution. Therefore, the Mann–Whitney *U* test was used, yielding a *p* value of 0.04, indicating a statistically significant difference (Z = −2.051). The effect size was *r* = 0.17, suggesting a small to medium effect.

## Discussion

This study analyzed children with MPP and pulmonary consolidation, using 3D CT reconstruction to calculate lung consolidation volume and its absorption rates after treatment. The results revealed that most children have 96% resolution within 2–3 weeks timeframe after effective treatment. Those who had a bronchoscopy may have a faster resolution rate but undertaking a flexible bronchoscopy under these circumstances is not a standard procedure in most settings.

The pathogenesis of lung inflammation and consolidation caused by MP remains unclear. Current research suggests it is primarily due to direct damage by the pathogen and abnormal immune responses (18, 19). MP infection is most common in preschool- and school-aged children. It has been reported that MPP with consolidation is more frequent in school-aged children than in preschoolers (20), likely because older children have a more mature T-lymphocyte response to alveolar macrophages, leading to higher cytokine production and more severe clinical manifestations (21). In this study, the mean age of the 238 children was 5 years and 11 months ± 2 years and 6 months, primarily in the preschool and school-age groups. Regarding sex differences, studies have found that MP infection rates are higher in females than males (21), potentially due to sex hormone differences, with females exhibiting stronger humoral and cellular immune responses than males (22). In our study, the male-to-female ratio was 109:129, which was also consistent with existing literature.

All children in this study presented with cough at admission, and most had fever (203 cases, 85.29%). These findings align with literature indicating that cough and fever are the primary clinical manifestations of pediatric MPP (23). Thus, it can be inferred that all children with MPP and pulmonary consolidation will exhibit cough, although fever is not universally present. Studies have also shown that fever duration correlates positively with MPP severity and is a risk factor for refractory MPP (24). In this study, the incidence of severe pneumonia was 34.87%, and respiratory failure was 7.56%, compared to the global incidence of severe community-acquired pneumonia (8%–33%) (25). These findings suggest that children with MPP and pulmonary consolidation have a slightly higher incidence of severe pneumonia but a relatively low incidence of respiratory failure. Laboratory findings revealed no significant increases in WBC or LDH levels, while hs-CRP levels showed mild elevation, indicating that these patients generally exhibit mild systemic inflammatory responses. Notably, current research highlights CRP and LDH as predictive factors for RMPP (26), suggesting that routine monitoring of these markers may help identify cases at risk of progression to RMPP.

The first-line treatment for MP infection remains macrolide antibiotics, with azithromycin being the most widely used. Studies have shown that macrolides not only possess broad-spectrum antibacterial activity but also exhibit immune-modulating effects independent of their antibacterial properties. These include anti-inflammatory effects, regulation of airway secretions, modulation of microbial immunity, and corticosteroid-sparing effects. However, their immunomodulatory effects typically require several weeks to manifest (27), whereas most children with MPP receive macrolides for less than one week.

In recent years, reports of macrolide resistance in MPP have increased. After the first isolation of MP strains from pediatric patients in 2000, macrolide resistance rates rose sharply from 5% in 2003 to 39% in 2008. In eastern countries such as Japan and China, resistance rates exceeded 80% after 2009, while western countries reported much lower rates: 8.2% in the United States, 0.9%–2.9% in Denmark, 3% in Germany, 10% in France, 26% in Italy, and 32% in Israel (28). Recent studies suggest that this rise in resistance is associated with genetic shifts in MP strains (e.g., P1 type 1 to type 2) and the widespread use of macrolides (7).

Glucocorticoids, known for their potent anti-inflammatory and immunoregulatory effects, can rapidly modulate immune cell activity, reduce excessive immune responses, improve microvascular circulation, and promote recovery. Studies indicate that glucocorticoids effectively shorten the duration of fever, cough, and lung crackles, alleviate inflammation, and improve treatment outcomes in severe MPP cases (29, 30). In this study, 15.12% of the 238 children required second-line antibiotics (doxycycline or levofloxacin), all beginning treatment in 2023, reflecting a rising trend in macrolide resistance in recent years.

CT-based 3D reconstruction is widely used in the diagnosis of pulmonary masses, accurately determining lesion size and location, and assessing their relationship with surrounding tissues (31). Among the study cohort, unilateral consolidation was more common than bilateral, with right-sided lesions (49.15%) more frequent than left-sided (37.39%), consistent with previous reports (32). The median initial lung consolidation proportion was 4.48%, slightly lower than the 5.17% reported in the literature (33). Upon follow-up CT after a median interval of 17 days (range 15–21 days), the median consolidation volume was 1.59 cc, with a median absorption rate of 96.47%. Complete absorption was observed in 20 cases, while only five cases showed absorption rates below 50%. These findings indicate that most pulmonary consolidations in children with MPP were largely absorbed within 2–3 weeks as confirmed by follow-up CT scans.

Comparing the BAL and non-BAL groups, significant differences were noted in variables such as fever duration at admission, hs-CRP levels, initial consolidation proportion, follow-up interval, and absorption rate. These findings may reflect the clinical practice of prioritizing BAL for patients with larger consolidations, as well as parental hesitance toward BAL under general anesthesia, particularly for younger children. After performing a 1:1 PSM, a comparison of the consolidation absorption speed between the two groups revealed a statistically significant difference (*P* < 0.05). The median absorption speed was 2.13 cc/d in the BAL group and 1.60 cc/d in the non-BAL group. Although BAL may moderately accelerate the resolution of pulmonary consolidation in pediatric MPP, the effect size remains small to moderate. Furthermore, performing flexible bronchoscopy in such clinical scenarios is not considered standard practice in most clinical settings. The decision to proceed with BAL should ultimately be determined through individualized clinical assessment and shared decision-making with parents or guardians.

According to relevant literature, the mechanism by which BAL promotes consolidation absorption in MPP involves direct clearance of mucus plugs—formed secondary to MP-induced respiratory epithelial damage, ciliary dysfunction, and excessive mucus secretion. By mechanically removing these obstructive plugs and inflammatory mediators via lavage, BAL restores airway patency, improves mucociliary clearance, facilitates lung re-expansion, and accelerates inflammatory resolution (13, 14).

Although BAL is generally believed to shorten the disease course and accelerate recovery, it is an invasive procedure requiring general anesthesia in children. Overuse may increase anesthesia-related risks, hospitalization costs, and potential harm to patients. Thus, it is crucial to carefully evaluate the indications for BAL in MPP with lung consolidation, posing a significant challenge for clinicians.

There are several limitations exist in this study. It is a retrospective analysis, and several subjective factors influenced the decision to perform bronchoscopy in children with MPP and pulmonary consolidation in real-world settings. These factors include the lack of clear clinical guidelines for bronchoscopy indications, variability in clinicians' judgment, parental education levels, financial concerns, anxiety about anesthesia risks, and acceptance of bronchoscopy. These factors may have contributed to sampling bias between the BAL and non-BAL groups. Additionally, the delineation of lung consolidation volumes in this study relied on manual operations. Although the measurements were averaged by two radiologists, some degree of error is inevitable. As a single-center study, the findings lack generalizability, highlighting the need for larger sample sizes and multi-center prospective studies in the future. Furthermore, this study focused solely on imaging-based follow-up of consolidation absorption and did not include follow-up data on pulmonary function. Limited research exists on the absorption process and mechanisms of pulmonary consolidation, highlighting the need for further studies to explore why MPP-related consolidation resolves within 2–3 weeks of effective treatment.

In conclusion, this study followed up on 238 children with MPP and pulmonary consolidation and found that using lung CT-based 3D reconstruction to evaluate consolidation changes, most children have 96% resolution within 2–3 weeks timeframe. Those who had a bronchoscopy may have a faster resolution rate but undertaking a flexible bronchoscopy under these circumstances is not a standard procedure in most settings.

## Data Availability

The original contributions presented in the study are included in the article/Supplementary Material, further inquiries can be directed to the corresponding author.
